# Toward an integrated governance framework for emerging contaminants

**DOI:** 10.1016/j.eehl.2026.100239

**Published:** 2026-04-07

**Authors:** Mingrong Liang, Shaokun Li, Yuling Liang, Meng Chen, Yunbo Song, Yongyue Lu, Lei Wang, Christian Sonne

**Affiliations:** a School of Biological Sciences, The University of Hong Kong, Hong Kong SAR, China; b Joint International Research Laboratory of Catastrophe Simulation and Systemic Risk Governance, Beijing Normal University, Zhuhai 519087, China; c School of National Safety and Emergency Management, Beijing Normal University, Beijing 100875, China; d College of Plant Protection, South China Agricultural University, Guangzhou 510642, China; e Department of Ecoscience, Aarhus University, DK-4000, Roskilde, Denmark

Emerging contaminants (ECs) encompass diverse hazards, including persistent chemicals, micro- and nanomaterials, and biologically mediated agents [1]. Their risks are often nonlinear, cumulative, and transboundary. Evidence already shows serious consequences across systems: phototoxic transformation products of common UV filters can kill corals [2]; pesticide-intensive landscapes are associated with marked declines in wild pollinators [3]; and chronic low-dose human exposure to persistent, bioaccumulative substances such as PFAS is linked to immunosuppression, cancer, and large annual economic losses [4]. The widespread detection of trifluoroacetic acid (TFA) in European cereal products further illustrates how persistent fluorinated breakdown products can accumulate through food systems [5]. At the same time, antimicrobial resistance, partly driven by environmental contamination pathways, contributes to millions of deaths each year and major macroeconomic costs [6]. EC governance is therefore not merely an environmental issue, but a cross-sector challenge spanning chemistry, ecology, public health, economics, and global policy.

In May 2022, China launched a new environmental management plan for chemicals, supported by an estimated US$300 million, to strengthen surveillance, monitoring, and risk assessment for priority ECs [1,7,8]. This signals a shift from reactive regulation toward anticipatory control. China, like other jurisdictions, continues to strengthen and optimize its governance systems to enable the reliable conversion of early warning into timely and enforceable risk reduction. The European Unionʼs REACH regime advances the principle of “no data, no market”, placing the burden of evidence generation on industry and enabling earlier-stage supply-chain screening [9]. In the United States, the Toxic Substances Control Act has expanded regulatory authority, but risk evaluation and management often continue to lag behind hazard identification [10]. More broadly, governance remains constrained by incomplete analytical coverage, fragmented institutional mandates, and weak disclosure requirements.

Closing this gap requires an integrated, lifecycle-based, data-driven, and enforceable governance framework. Such a system should connect high-resolution monitoring, interoperable cross-agency data platforms, mandatory supply-chain transparency, and uncertainty-aware risk evaluation. A global minimum dataset, harmonized monitoring standards, adaptive assessment protocols, and rapid-response regulatory tools are necessary to translate detection into intervention. Advanced analytics can improve prioritization, but measurable reductions in exposure and harm will depend on coordinated institutions, safer substitution, clear accountability, and sustained implementation capacity. Ultimately, governance should be judged not only by how well contaminants are detected, but also by how effectively systemic risks are reduced.

## CRediT authorship contribution statement

**Mingrong Liang:** Conceptualization, Methodology, Data curation, Formal analysis, Project administration, Resources, Supervision, Validation, Visualization, Writing – original draft, Writing – review & editing. **Shaokun Li:** Conceptualization, Writing – original draft, Writing – review & editing. **Yuling Liang:** Conceptualization, Writing – original draft, Writing – review & editing. **Meng Chen:** Conceptualization, Writing – original draft, Writing – review & editing. **Yunbo Song:** Conceptualization, Writing – original draft, Writing – review & editing. **Yongyue Lu:** Conceptualization, Supervision, Writing – original draft, Writing – review & editing. **Lei Wang:** Supervision, Writing – original draft, Writing – review & editing. **Christian Sonne:** Conceptualization, Supervision, Writing – original draft, Writing – review & editing.

## Declaration of competing interest

The authors declare no conflict of interest.
